# Colloid Cyst at the Foramen of Monro Leading to Symptomatic Obstructive Hydrocephalus

**DOI:** 10.51894/001c.6980

**Published:** 2018-09-26

**Authors:** Pratik Shah, Joseph Flynn

**Affiliations:** 1 PGY2 Department of Emergency Medicine McLaren Macomb Hospital; 2 Department of Emergency Medicine McLaren Macomb Hospital

**Keywords:** emergency neurology, hydrocephalus, foramen of monro, colloid cyst

## Abstract

A Caucasian female in her late forties presented to the Emergency Department (ED) with headache, ataxia, and mental status changes. A CT brain demonstrated dilated lateral ventricles with transependymal edema. An MRI of the brain demonstrated marked obstructive hydrocephalus from an obstructing colloid cyst at the level of her Foramen of Monro. The patient was transferred to a tertiary care center for neurosurgical removal of the cyst. Three months later, the patient was doing well and had resumed all activities of daily living without any residual neurological deficits. The goal of this case report is to educate readers on this atypical presentation of hydrocephalus, its symptomatology, and management to allow physicians to be more comfortable in making treatment decisions.

## INTRODUCTION

A colloid cyst is considered a rare developmental malformation in the brain and not a true neoplasm, as they are benign masses, filled with proteinaceous fluid. Colloid cysts make up between 0.5% to 1% of intracranial tumors.^1^ Although rare, colloid cysts can have significant neurologic sequelae due to ventriculomegaly (i.e., a condition of the brain that can occur in the fetus wherein the lateral ventricles become dilated) caused by the mass effect, even on presentation to the ED.

## METHODS

### Case Report

A Caucasian female in her late forties drove to our ED with a chief complaint of headache. Her headache had been persistent over the past eight days. She characterized it as a constant, throbbing sensation localized to the frontal aspect of her head. She admitted to experiencing prior migraine headaches, however, she noted that this particular instance felt different. The patient denied maximal intensity upon onset of symptoms. She denied photophobia, phonophobia, or any other neurological deficits. When her headache started, she had a concurrent upper respiratory infection. She was prescribed Promethazine 25 mg PO at an urgent care facility. According to patient’s boyfriend, ever since she started taking Promethazine, she had been acting “different.”

The night before her arrival to our ED, the patient had worked a long shift as a valet driver. Upon arriving home, she took a dose of Promethazine and fell asleep. Upon arriving to work the following morning, her coworkers noted that her car had large dents on the front bumper. When she got out of the car, she was wearing a short skirt and mismatched high heels despite the frigid outside temperatures. She was ataxic and speaking incoherently and was sent to our ED for evaluation.

Upon arrival to the ED, the patient had stable vital signs. Her medical history was significant for hypertension. Her physical examination was unremarkable apart from neurological examination. When asked the date, the patient responded “October 1967.” Her head appeared normocephalic. There were no meningeal signs. Her speech was incoherent. Finger to nose testing was dysmetric with tendency to overshoot. Additionally, she was ataxic with broad gait.

Considerations were given to possible adverse drug reaction to Promethazine, other toxic or infectious etiologies, or structural lesions. Laboratory studies were within normal limits. While waiting to receive a CT scan of her brain, a bedside ocular ultrasound was performed which demonstrated an optic nerve sheath diameter less than 5 mm. without evidence of papilledema. Her CT brain without IV contrast demonstrated dilated lateral ventricles with associated transependymal edema. (Figure 1) An emergent MRI of the brain without contrast was completed to identify a colloid cyst at the level of the Foramen of Monro measuring 1.3 x 1.3 cm. (Figure 2) There was marked obstructive hydrocephalus and cerebral sulcal effacement seen in the image consistent with cerebral edema. The patient was admitted to our hospital for evaluation by the Neurology and Neurosurgery teams.

**Figure 1 A:**
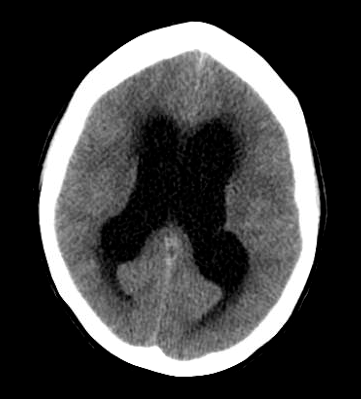
CT of the Brain without Contrast A: Axial image demonstrating dilated lateral ventricles with transependymal edema

**Figure 1 B:**
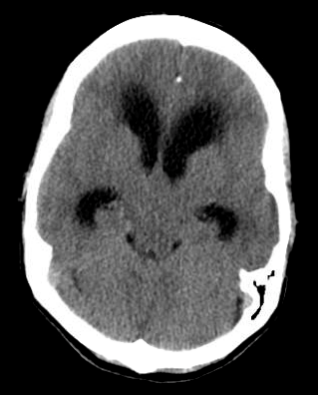
CT of the Brain without Contrast B: Axial image demonstrating possible obstructive pathology at the level of the Foramen of Monro

**Figure 2 A:**
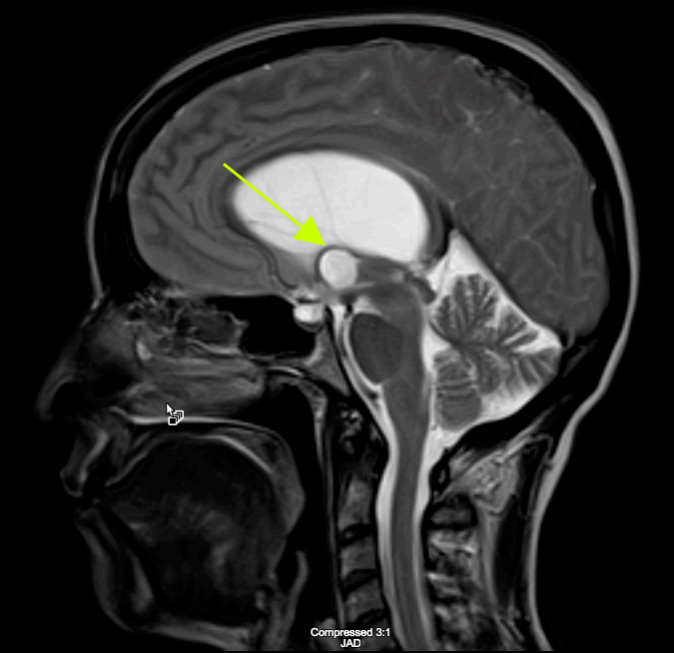
MRI of the Brain without Contrast A: T2 weighted sagittal image demonstrating a colloid cyst measuring 1.3 cm by 1.3 cm at the level of the Foramen of Monro.

**Figure 2 B:**
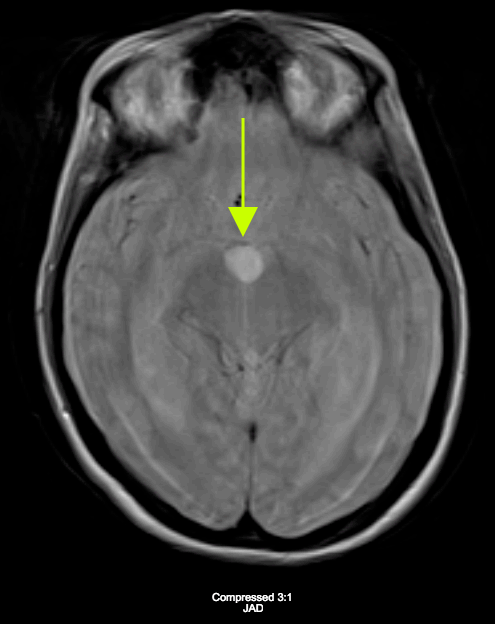
MRI of the Brain without Contrast B: FLAIR image displaying hyperintense signal at the Foramen of Monro consistent with colloid cyst.

The Neurosurgery service recommended transferring the patient to a tertiary care facility for open craniotomy and resection of the colloid cyst. At the tertiary care hospital, a right craniotomy was performed. A colloid cyst was identified at the roof of the third ventricle extending into the lateral ventricles through the Foramen of Monro. The cyst was subsequently dissected out. An external ventricular drain (EVD) was kept at the patient’s Foramen of Monro. The EVD device was removed at 2-week follow-up. On interview with the patient three months following surgery, she was feeling much better and has resumed employment as a valet driver.

## DISCUSSION

A colloid cyst has a wall lined with epithelial and goblet cells secreting protein-rich fluid.^2^ While this condition is considered a rare developmental malformation and not a true neoplasm, it does occur in approximately between 0.5% to 1% of intracranial tumors.^1^ Typically, this type of cyst can occur at any age but people generally do not become symptomatic until the third to fifth decades of life.^3^ In 99% of cases, it is found posterior to the Foramen of Monro and arises from the anterior roof of the third ventricle.^4,5^ At the level of the Foramen of Monro, the cyst may prevent drainage of cerebrospinal fluid from the lateral ventricles to the third ventricle. In effect, hydrocephalus and resulting transependymal edema from the increased intracranial pressure (ICP) may be seen, as was the case for this patient. Colloid cysts carry a risk of mortality ranging from 3.1 to 10%.^4,6^

Depending on the extent of ventriculomegaly, patients may present with neurological symptoms ranging from headaches to herniation. The triad of “wet, wacky and wobbly” is often found in medical school exams, all of which can be found in patients with hydrocephalus associated with obstructing colloid cysts.^7,8^ Papilledema may also be present due to ICP causing pressure on the optic nerve sheath.^9^ For this reason, it is important to check the patient’s vision and test the optic nerve with visual acuity and visual field testing.

In a study of 155 patients with newly diagnosed colloid cysts, Pollock et al. discovered that symptomatic colloid cysts were associated with 4 main variables: patient age, cyst size, ventricular dilation, and signal strength on T2 weighted MRI. Younger patients, 44 years old versus 57 years old (p < 0.001) were more symptomatic. Patients with larger cyst size, 13 mm. versus 8 mm. (p < 0.001) were also more symptomatic. Patients with increased ventricular dilation of 83% versus 31% (p < 0.001) also displayed more symptoms. Finally, increased signal on T2-weighted MRI, 44% versus 8% (p = 0.001) tended to be more symptomatic. The most significant variable of these was ventriculomegaly. Patients with larger ventricles were more symptomatic.^10^

In 2016, Beaumont et al. published the Colloid Cyst Risk Score (CCRS), a method used to stratify the risk of a patient to develop obstructive hydrocephalus and guide physicians to choose appropriate treatment pathways. Patients with a CCRS ≥ 4 are considered at high risk for lesion progression and obstructive hydrocephalus. One point is given for age below 65 years, one point for presence of headache, one point for axial diameter of cyst >7 mm., one point for FLAIR (i.e., fluid attenuated inversion recovery) hyperintensity on MRI, and one point for location of the colloid cyst in the risk zone. According to this scale, our patient would have been assigned a CCRS of 5 and did in fact have obstructive hydrocephalus requiring surgery.

Imaging studies are essential in the workup of patients presenting with symptoms suggestive of possible hydrocephalus. A CT brain without contrast is the initial test of choice. Approximately 2/3 of colloid cysts appear hyperdense on imaging. The lesions are usually round or ovoid and are well delineated. Most cysts range between 5-25 mm.^10^ The appearance of colloid cysts on MRI is important for neurosurgeons, because the surgical success rate is lower in colloid cysts that have decreased MRI T2-signal intensity.^11^ It is also important to keep in mind that lumbar puncture can be fatal as non-draining obstructive hydrocephalus can lead to herniation. An ophthalmologic evaluation may be useful if diplopia is a presenting complaint or if papilledema is found on physical examination as this may be a marker for increased ICP.^9^

Various management options exist depending on the severity of a patient’s symptoms. For patients who are symptomatic and have a higher degree of ventriculomegaly, more immediate surgical options include craniotomy for microsurgical resection, neuroendoscopic removal, and cerebrospinal fluid diversion with ventriculoperitoneal (VP) shunts.^12,13^ In our patient’s case, she did have symptomatic hydrocephalus as a result of the obstructing colloid cyst and underwent a craniotomy. In asymptomatic patients, conservative management with serial MRIs is preferred.^12^

Microsurgical resection through the transcortical-transventricular or transcallosal approach is considered the standard of surgical treatment for symptomatic patients.^13^ The transcortical approach involves a craniectomy over the middle frontal gyrus. This transcortical approach carries an increased risk of postoperative epilepsy. Other possible complications include hematoma, memory deficits, mutism, and hemiplegia.^14^

## CONCLUSIONS

Although rare, colloid cysts can have significant neurologic sequelae as seen in this patient’s case. The case reported here was a unique presentation to the ED given her age and significant ventriculomegaly from obstructing hydrocephalus. She presented with persistent headache, altered mental status, and ataxia. Her MRI displayed a colloid cyst at the level of the Foramen of Monro that was surgically dissected. The goal of this case report is to familiarize readers with the presentation, symptomatology, and management associated with colloid cysts. After reading this report, we hope that clinicians will consider this condition as a differential diagnosis in those patients who present with acute neurologic changes similar to our patient.

The authors report no external funding source for this study.

The authors declare no conflict of interest.

Submitted for publication March 2018.

Accepted for publication July 2018.
